# Metabolomic and biochemometric profiling of *Tecoma stans* with mechanistic insights of its in vitro cytotoxic activity

**DOI:** 10.1038/s41598-026-67317-z

**Published:** 2026-08-27

**Authors:** Ahmed Sekkien, Noha Swilam, Dalia Adel Al-Mahdy, Maha R. A. Abdollah, Mohamed Mohey Elmazar, Meselhy R. Meselhy, Muhammad A. Alsherbiny

**Affiliations:** 1 Department of Pharmacognosy, Faculty of Pharmacy, The British University in Egypt (BUE), Cairo, 11837 Egypt; 2 Department of Pharmacognosy, Faculty of Pharmacy, Cairo University, Kasr El Aini St, Cairo, 11562 Egypt; 3 Department of Pharmacognosy, Faculty of Pharmacy, Modern University for Technology and Information, Cairo, Egypt; 4 Department of Pharmacology, Faculty of Pharmacy, The British University in Egypt (BUE), Cairo, 11837 Egypt; 5 Innovation Centre, Victor Chang Cardiac Research Institute, Darlinghurst, NSW 2010 Australia

**Keywords:** Bignoniaceae, Chrysoeriol, Molecular networking, LC-MS/MS, Apoptosis, Ovarian carcinoma, Biochemistry, Cancer, Cell biology, Chemical biology, Drug discovery, Plant sciences

## Abstract

*Tecoma stans* is an ornamental plant recognized for its diverse biological activities, including notable cytotoxic effects. This study integrates LC–MS/MS-guided biochemometric analysis with mechanistic cytotoxicity evaluation to prioritize metabolites potentially associated with the cytotoxic activity of *T. stans* leaves. The hydroalcoholic extract and its solvent fractions *n*-hexane (HEX), dichloromethane (DCM), and ethyl acetate (EAC) were assessed for in vitro cytotoxicity against human lung adenocarcinoma (A549) and ovarian carcinoma (SKOV-3) cell lines using the MTT assay. Comprehensive LC–MS/MS-based metabolomic profiling and biochemometric correlation analysis were performed on the DCM fraction. The major correlated compound, chrysoeriol (CRY), was isolated and, along with the DCM fraction, subjected to cell cycle analysis, apoptosis assays, scratch wound healing assays, and ELISA-based quantification of apoptotic and metastatic markers (caspase-3, p-STAT3, Bcl-2, and MMP-2) in SKOV-3 cells. The DCM fraction exhibited the highest potency, particularly against SKOV-3 cells (IC₅₀ = 18.50 µg/mL). Metabolomic profiling led to the annotation of 107 metabolites, where CRY, α-sulfoquinovosyl monoacylglyceride (α-SQMG), tecomanine, and hydroxyskytanthine were prioritized as metabolites potentially associated with cytotoxic activity. DCM induced pronounced sub-G₁ arrest (71.42%, *P* < 0.0005) and increased late apoptosis to 23.32%, whereas CRY elevated sub-G₁ accumulation to 48.02% and late apoptosis to 12.22% (*P* < 0.0005). Furthermore, both treatments markedly reduced scratch wound closure and modulated key signaling pathways through caspase-3 activation and suppression of p-STAT3, Bcl-2, and MMP-2. The CRY and DCM fraction of *T. stans* leaves exerted their cytotoxic effects against SKOV-3 cells through the induction of apoptosis, wound closure inhibition and modulation of key oncogenic proteins. This study provides the first biochemometric and mechanistic validation of *T. stans* leaves DCM fraction and CRY in SKOV-3 cells, highlighting the potential combined contribution of its phytochemical constituents.

**Supplementary Information:**

The online version contains supplementary material available at https://doi.org/10.1038/s41598-026-67317-z.

## Introduction

*Tecoma stans* (L.) Juss. ex Kunth, a member of the family Bignoniaceae, is an ornamental shrub native to Latin America and widely cultivated in tropical and subtropical regions^1^. Beyond it’s ornamental value, *T. stans* has been traditionally used to treat various diseases. Its leaves and flowers have been traditionally used in the treatment of hyperglycemia, dysentery, jaundice, headache, and renal disorders, and have also been employed as eupeptic agents and mild tonics. In addition, the bark has been reported to exhibit smooth muscle relaxant, mild cardiotonic, and choleretic properties, while the seeds have been traditionally utilized in the management of piles^2^.

Furthermore, extracts from leaves and flowers of *T. stans* have demonstrated a broad spectrum of biological activities, including antidiabetic, antimicrobial, and antioxidant properties^2,3^. Several studies have also demonstrated the pronounced cytotoxic potential of *T.stans* against multiple cancer cell models, with frequent emphasis on breast (MCF-7 and MDA-MB-231)^4,5^, liver (HepG2), lung (A549)^6^, and cervical (HeLa)^7^ cell lines, alongside additional models including laryngeal carcinoma (HEP-2) ^8^, rhabdomyosarcoma (RD-CCL 136)^9^, prostate cancer (PC-3), colorectal cancer (HCT-116 and SW-480), melanoma (SK-MEL-28) and bladder cancer (T24)^5,10^.

Previous phytochemical investigations have revealed a complex array of secondary metabolites, such as flavonoids, alkaloids, phenylethanoids, phenylpropanoids, lignans, phenolic acids, terpenoids, volatile constituents, and fatty acids^7,11,12^, many of which possess broad cytotoxic properties^13–15^. However, the contribution of specific metabolites to this activity and the underlying molecular mechanisms remain insufficiently investigated. Therefore, LC-MS-driven metabolomic profiling coupled with biochemometric analysis is warranted to identify bioactive metabolites without ignoring the less abundant metabolites, which are always overlooked in bioactivity-guided fractionation^16,17^.

In the same context, lung and ovarian cancers continue to cause major global health burdens. Where lung cancer remains the leading cause of cancer-related mortality worldwide, with an estimated 2.21 million new cases and 1.80 million deaths reported in 2020 ^18^. Likewise, ovarian cancer represents one of the most lethal gynecological malignancies, largely due to late diagnosis and the frequent emergence of chemoresistance^19^. Globally, more than 324,000 new ovarian cancer cases were reported in 2022, and the associated mortality is expected to increase substantially by 2050 ^20^. Given the previously reported cytotoxic activity of *T. stans* extracts against several cancer cell models, the leaves, which have been previously investigated for cytotoxic activity and are readily available throughout the year, were selected for the present study. In the same context, the A549 cell line used as a widely used model for non-small cell lung cancer and commonly employed to evaluate anticancer agents^21^, and the ovarian carcinoma cell line SKOV-3, an established model characterized by aggressive and metastatic behavior^22,23^, were both selected to evaluate the anticancer potential of *T. stans.*

Accordingly, the present study aimed to a)- evaluate the cytotoxic activity of *T. stans* leaf extract and its *n*-hexane (HEX), dichloromethane (DCM) and ethyl acetate (EAC) fractions against the ovarian carcinoma cell line (SKOV-3) and Human lung carcinoma cell line (A-549), and b)- elucidate the mechanistic basis of cytotoxic activity of the most potent fraction and chrysoeriol (CRY) as one of its major compounds through a series of cellular and molecular assays. In parallel, LC–MS/MS-based metabolomic profiling coupled with biochemometric analysis was employed to characterize the phytochemical composition of the crude extract and fractions and to identify key metabolites contributing to the observed cytotoxic effects. To our knowledge, this represents the first metabolomics-guided biochemometric investigation linking *T. stans* metabolites to mechanistically validated cytotoxicity in SKOV-3 cells. Furthermore, this study provides the first mechanistic evidence for the cytotoxic activity of chrysoeriol in ovarian carcinoma.

## Methods

### Chemicals and reagents

All chemicals used in this study for extraction, fractionation, isolation, and LC-MS/MS were of HPLC or analytical grade and were procured from Fisher Scientific (Loughborough, United Kingdom).

Cell culture media, including Dulbecco’s Modified Eagle Medium (DMEM), fetal bovine serum (FBS), penicillin, and streptomycin, were obtained from Nawah scientific (Cairo, Egypt). Additional reagents, including MTT, phosphate-buffered saline (PBS), dimethyl sulfoxide (DMSO), and trypsin-EDTA, were also obtained from Nawah Scientific (Cairo, Egypt).

### Plant material

The Fresh leaves of *Tecoma stans* (L.) Juss. ex Kunth were collected in August at the flowering stage from the gardens of the British University in Egypt (BUE). The taxonomic identity of the material was confirmed by Dr. Abdelhalim Abdel Mogally, Botanical Taxonomist, Flora and Phyto-Taxonomy Department, Horticultural Research Institute, Dokki, Giza, Egypt. A voucher specimen (11-9-25-F) was deposited in the Herbarium of the Pharmacognosy Department, Faculty of Pharmacy, Cairo University, and is maintained as a permanent reference for this study. All experimental research and field studies involving plant material were conducted in accordance with relevant institutional, national, and international guidelines and legislation; *Tecoma stans* is not a protected or endangered species, and its collection from the BUE campus did not require specific permits or ethical approval.

### Extraction and fractionation

A 3.5 kg of air-dried *T. stans* leaves were powdered and subjected to extraction by maceration in 70% ethanol (15 L) at room temperature for 24 h, followed by filtration. The process was repeated for four more days until exhaustion. The filtrates were combined and concentrated under reduced pressure to give 650 g of dry residue (TOT).

Part of TOT (350 g) was suspended in distilled water (1.5 L) and successively partitioned with 5 L of organic solvents with increasing polarity: *n*-hexane (HEX), dichloromethane (DCM), and ethyl acetate (EAC) to yield, after evaporation 29 g, 21 g, and 52 g of dry residues of HEX, DCM, and EAC fractions, respectively.

### Isolation of chrysoeriol (CRY)

A portion of the DCM fraction (10 g) was subjected to silica gel column chromatography for compound isolation. The fraction was loaded onto a glass column packed with silica gel 60 (70–230 mesh, Merck, Germany) and eluted using a stepwise gradient of n-hexane - ethyl acetate (EtOAc) with increasing polarity. Fractions of approximately 50 mL were collected and monitored by thin layer chromatography (TLC) on silica gel plates (silica gel 60 F254, Merck) using n-hexane - EtOAc (7:3, v/v) as the mobile phase. Spots were visualized under UV light at 254 and 365 nm. This process resulted in the isolation of chrysoeriol (CRY) as a yellow amorphous powder with a total yield of 428 mg from the DCM fraction.

### LC-MS/MS metabolomic and biochemometric analyses

The samples were dissolved in methanol (10 mg mL^− 1^) and filtered through a 0.2 μm PTFE filter for further UPLC-MS analyses. The analysis was performed using ultraperformance liquid chromatography coupled with electrospray ionization quadrupole time of flight tandem mass spectrometry (UPLC–ESI–QTOF–MS/MS) via Agilent 1290 UPLC system interfaced with an Agilent 6546 quadrupole time-of-flight mass spectrometer with dual AJS electrospray ion source (ESI). Agilent MassHunter Data Acquisition was used for data acquisition B09.00. The samples were analyzed in positive and negative modes utilizing a data dependent “AutoMSMS” acquisition with the following parameters. Narrow isolation width MS/MS of ~ 1.3 amu, 4 maximum precursors per cycle with activated active exclusion after 2 spectra for 0.2 min. MS and MSMS range of 50-1200 *m/z* were implemented with fixed collision energies at 10, 20, and 40 eV. Gas and sheath gas temperatures were set at 320 and 350 °C, respectively, with a 10 L/min gas flow and 35 L/min sheath gas flow, together with 3500–4500 V for capillary voltage in negative and positive modes. Other source parameters, such as the fragmentor, skimmer1, and octupole RFPeak, were set to 120, 65, and 750, respectively, along with 1000 V nozzle voltage. Agilent TOF reference mass solution kit (G1969-85001) was simultaneously infused to calibrate the masses.

Chromatographic separation was performed with an injection volume of 5 µL and A 0.28 ml/min flow rate. Water and methanol were used as mobile phases A and B, respectively, with 0.2% formic acid. The following gradient was used; 0% B was kept for 1 min, inclined to 80% B at 16 min, kept for 2 min, then declined to 0% B at 18.1 min, followed by 2 min conditioning. ACQUITY UPLC HSS-T3 Column (1.8 μm, 2.1 × 100 mm, Waters Corporation, Milford, USA) with a 2.1 × 5 mm T3 VanGurd™ PreColumn (Waters Corporation, Milford, USA) was used, and the column temperature was kept at 45^◦^C.

Raw data were processed with MSDIAL version 5.1, where negative and positive mode batches were processed separately using 0.01 and 0.025 Da for MS^1^ and MS^2^ tolerances, 0.1 Da mass slice width, 1000 amplitude minimum peak height and aligned to a pooled quality control reference (QC) with 0.05 min RT tolerance and 0.015 Da MS^1^ tolerance^24^. The Riken public spectral library version S17 was initially implemented during MSDIAL processing for metabolite identification. MSDIAL-exported features were further cleaned and processed using MS-CleanR with a minimum blank ratio set to 0.8 and a maximum relative standard deviation (RSD) set to 30 ^25^. The MSCleanR-retained features were annotated with MS-FINDER version 3.52 ^26^. The MS1 and MS2 tolerances were set to 5 and 15 ppm, respectively, with a 1% relative abundance cut-off. The formula finder was processed with C, H, O, P, S and N atoms with 20% isotopic ratio tolerance. MSFinder mined the filtered compounds based on exact mass and fragmentation using generic databases included in MS-FINDER (i.e.,Universal Natural Product Database (UNPD), Collection of Open Natural Products (COCONUT), Human Metabolome Database (HMDB), food database (FooDB), Chemical Entities of Biological Interest (ChEBI), and LIPID MAPS. Feature filtration strategies were furtherly adopted to retain the major and differential metabolites (Top differentials based on OPLSDA S-Plot tagged as ‘D’ in the supplementary sheets to tag features with Correlation ≤ 0.95 and P1 ≤ -25 with FC > 2 of each extract/fraction compared with TOT^16,27,28^. The statistically significant features with absolute fold change ≥ 2 and Adjusted P (FDR) ≤ 0.05 against blank samples were kept. Metaboanalyst 5.0 and SIMCA 15.0.2 (Sartorius Stedium Data Analytics AB, Sweden) was used for statistical testing and multivariate data visualisation and filtering. Features associated with cytotoxic activity were prioritized based on a correlation coefficient > 0.9 and an adjusted *P*-value (Q) ≤ 0.05, which were used as stringent filtering criteria to reduce false-positive associations. Differential and major compounds along with compounds positively correlated to activity via bioactive molecular network workflow^17^(https://github.com/DorresteinLaboratory/Bioactive_Molecular_Networks*)* to correlate the compound to the IC_50_ against SKOV 3 and A549 cancer cell lines were thoroughly checked for their tentative ID using Sirius version 6 ^29^. Sirius implements BUDDY^30^ for bottom up molecular formula generation, NPclassifier^31^ and ClassFire^32^ via CANOPUS^33^ for compounds classification, CSI: Finger ID^34^ and MSNovelist^35^ for denovo structure generation from mass spectra (Table S1).

### Cell culture

SKOV-3 and A549 cell lines were obtained from Nawah scientific (Cairo, Egypt). Cells were maintained in Dulbecco’s Modified Eagle Medium (DMEM) or RPMI-1640, supplemented with 10% heat-inactivated fetal bovine serum (FBS), 100 U/mL penicillin, and 100 µg/mL streptomycin. Cultures were incubated at 37 °C in a humidified atmosphere containing 5% CO₂ and passaged at approximately 80% confluence. All cell handling, including seeding, medium changes, and subculturing, was performed under aseptic conditions in a Class II biosafety cabinet. Cells were used for experiments during the logarithmic growth phase^36^.

### Standardization of DCM fraction using UPLC-PDA

Quantitative analysis of chrysoeriol (CRY) in the DCM fraction was performed using a Thermo Fisher UPLC Dionex Ultimate 3000 system (Agilent, Santa Clara, CA, USA) equipped with a PDA detector and a Hypersil Gold™ C18 column (250 × 4.6 mm, 3 μm particle size). The mobile phase consisted of solvent A (0.1% acetic acid in water) and solvent B (acetonitrile). The gradient program was initiated at 100% A and 0% B, followed by a linear increase to 100% B over 15 min. The flow rate was maintained at 0.9 ml/min and the column temperature was set at 30 °C. UV detection was carried out at 345 nm. A calibration curve for CRY was constructed over a concentration range of 62.5–1000 µg/mL.

### MTT cytotoxicity assay

The cytotoxic activity of extract, fractions, and isolated compounds was assessed using a modified 3-(4,5-dimethylthiazol-2-yl)-2,5-diphenyltetrazolium bromide (MTT) assay as described by Mosmann (1983). Stock solutions of the test samples were prepared in 10% DMSO and further diluted with complete medium to the required concentrations. Cells were seeded into 96-well plates at 3 × 10^4^ cells/well and allowed to adhere overnight. The following day, cells were treated in triplicate with serial dilutions (3.906–1000 µg/mL) and incubated for 72 h. After treatment, wells were washed three times with sterile PBS, followed by the addition of MTT reagent (20 µL; 5 mg/mL in PBS) and incubation for 4 h at 37 °C. Formazan crystals were dissolved in 200 µL of acidified isopropanol (0.04 M HCl), and absorbance was measured at 540 nm with a reference wavelength of 620 nm using a BMG LABTECH^®^ FLUOstar Omega microplate reader (Ortenberg, Germany). Cell viability was calculated relative to vehicle treated controls using the equation below, and IC₅₀ values were determined by non-linear regression using GraphPad Prism v9.0 (GraphPad Software Inc., USA)^37^.

$$\:\%\:viability=\left(\frac{{Absorbance}_{treated}-{Absorbance}_{blank}}{{Absorbance}_{control}-{Absorbance}_{blank}}\right)\times\:100$$

### Cell cycle analysis

Following treatment with the DCM, CRY and doxorubicin by 48 h, SKOV-3 cells (~ 1 × 10⁵) were harvested by trypsinization, washed twice with ice-cold phosphate-buffered saline (PBS, pH 7.4), and fixed in 2 mL of 60% ice-cold ethanol for 1 h at 4 °C. Fixed cells were washed twice with PBS and resuspended in 1 mL PBS containing RNase A (50 µg/mL) and propidium iodide (PI; 10 µg/mL). After 20 min incubation at 37 °C in the dark, DNA content was analyzed using an ACEA NovoCyte™ flow cytometer (Agilent Technologies, Santa Clara, CA, USA) with FL2 channel detection (λ_ex/em = 535/617 nm). A total of 12,000 events were acquired per sample, and phase distribution (G₀/G₁, S, G₂/M, and sub-G₁) was calculated using NovoExpress™ software version 1.1.0 ^38^. Representative gating strategies for the cell-cycle and Annexin V/PI assays are provided in Supplementary Fig. S12.

### Apoptosis assay

Apoptotic and necrotic cell populations were quantified using the Annexin V-FITC Apoptosis detection Kit (Abcam, UK) and 2 fluorescent channels flowcytometry. Treated SKOV-3 cells (~ 1 × 10⁵) were washed twice with ice cold PBS, stained with 0.5 mL Annexin V-FITC/PI solution, and incubated for 30 min at room temperature in the dark. Samples were analyzed immediately using the ACEA NovoCyte™ flow cytometer with FL1 (Annexin V-FITC, λ_ex/em = 488/530 nm) and FL2 (PI, λ_ex/em = 535/617 nm) channels, acquiring 12,000 events per sample. Data were processed by quadrant analysis after gating the intact cell population with exclusion of debris and subcellular events to distinguish viable, early apoptotic, late apoptotic, and necrotic cells^38^. The representative gating strategy for Annexin V/PI quadrant analysis is provided in Supplementary Fig. S12.

### Scratch wound healing assay

Cell migration was assessed by creating a uniform scratch in confluent SKOV-3 monolayers seeded at 2 × 10^5^ cells/well in 12 well plates. After incubation overnight in DMEM with 5% FBS, scratches were introduced using a sterile 200 µL pipette tip, and wells were washed with PBS to remove debris. Control wells received fresh complete medium, while treatment wells received fresh complete medium containing the test fraction, CRY, or doxorubicin. Wound closure was monitored at 0, 24, 48, 72, 96, and 120 h using an inverted phase contrast microscope. Images were taken using MII ImageView software v3.7 (Microscopes International, USA), and wound widths were measured using Fiji-ImageJ public domain software (NIH, Bethesda, MD)^39^.

### ELISA quantification of apoptotic and metastatic markers

SKOV-3 cells (5 × 10⁶) were seeded in T-75 flasks and allowed to adhere for 48 h. After treatment with tested fraction and compounds for 72 h, both adherent and floating cells were collected and processed according to the manufacturers’ recommended protocols for cell lysate preparation. Cells were washed twice with cold PBS and lysed in 100 µL RIPA buffer (Thermo Scientific, USA) supplemented with protease and phosphatase inhibitor cocktails (HALT™, Thermo Scientific). Lysates were incubated at 4 °C for 30 min with gentle agitation, centrifuged at 280 × g for 10 min at 4 °C, and the resulting supernatants were collected for immediate use or stored at − 80 °C for subsequent ELISA analysis.^38^.

Next, the protein levels of Caspase-3, p-STAT3, Bcl-2 and MMP-2 were determined using Caspase-3 (Active) ELISA Kit (Catalog No. KHO1091, Invitrogen, USA), Phosphotyrosine STAT3 ELISA Kit (Catalog No. ab279941, Abcam, UK), Zymed^®^ Human Bcl-2 ELISA Kit (Catalog No. 99 − 0042, Invitrogen/Zymed, USA) and MMP-2 Human ELISA Kit (Catalog No. ab100606, Abcam, UK), respectively, as described by the manufacturers’ protocols. For all ELISAs, absorbance was measured at 450 nm using a microplate reader, and concentrations were interpolated from standard curves.

###  Statistical analysis

All experiments were repeated at least three times with three independent biological experiments (*n* = 3) per treatment, representative data are shown. Statistical analyses were performed using these biological replicates, and data are presented as mean ± SD. Statistical comparisons between groups were conducted using one-way or two-way analysis of variance (ANOVA), followed by Tukey’s post hoc test. An adjusted p-value < 0.05 was considered statistically significant. All statistical analyses and graphical visualizations were performed using GraphPad Prism software, version 9.0. (GraphPad Software Inc., La Jolla, CA, USA).

### Declaration of generative artificial Intelligence (AI) and AI assisted technologies

The authors acknowledge the use of generative artificial intelligence (ChatGPT-5) in refining the language and readability of specific sections of this manuscript. All outputs produced by this tool were carefully reviewed and substantially edited by the authors as needed. The authors take full responsibility for the content and conclusions of the final published work.

## Results

### UPLC–ESI–QTOF–MS/MS profiling of *T. stans* metabolites

The phytochemical profile and data analysis of *T. stans* extract and fractions led to the tentative identification of 107 metabolite (56 were detected in positive ion mode and 51 in the negative ion mode).

The annotated metabolites represent a diverse set of chemical classes including, shikimates and phenylpropanoids (27 compounds) followed by fatty acids and lipid derivatives (26 compounds), alkaloids (24 compounds), terpenoids (20 compounds), in addition to, amino acids, polyketides and carbohydrates (10 compounds) (Table 1 and Table S1).

This class distribution mirrors the mixed polarity of the fraction set and the complementary ionization behavior in positive and negative modes where the neutral and weakly basic species tended to be annotated via [M + H]^+^ or [M + Na]^+^ in positive ESI, whereas acidic and glycosylated metabolites were commonly observed as [M–H]^–^ or [M+formate]^–^ in negative ionization mode.

Among the annotated metabolites, shikimate derived and phenylpropanoids constituted a prominent class, including major constituents such as luteolin [2005], chrysoeriol [2207], apigenin [1816], scroside D [7472], acteoside [7066], isoacteoside [7065], and umbelliferone [P_1671], highlighting the prevalence of oxygenated aromatic metabolites, particularly flavonoidal and caffeoyl-containing derivatives.

Alkaloids also formed a major group, with representative metabolites including tecomanine [P_2074], hydroxyskytanthine [P_2188], tecostanine [P_2190], and dehydroskytanthine [P_1760], consistent with the characteristic alkaloidal profile previously reported for *T. stans*.

In addition, fatty acids and related lipid derivatives were well represented, including 13-hydroxyoctadeca-9,11,15-trienoic acid (13-HOTrE) [2131], stearidonic acid [P_4357], monolinolenin [P_6456], and sulfoquinovosyl monoacylglyceride (SQMG) [6089], indicating the occurrence of both free fatty acids and structurally modified lipid derivatives such as monoacylglycerols and sulfolipids. Terpenoids were also detected in both ionization modes, with predominant representation in the positive mode of the n-hexane fraction, and included major constituents such as citronellal [P_1150], D-citronellol [P_1149], and tecoside [P_10715], indicating the presence of both volatile terpenoids and glycosylated terpenoid derivatives.

The overlaid base peak chromatograms of the TOT and it’s fractions in both positive and negative ionization modes have been added to the Supplementary file (Fig. S1 and Fig S2).

**Table 1:** Metabolites identified from *T.stans* extract and fraction in positive and negative ionisation modes.

Cluster ID	RT (min)	m/z	Adduct	Formula	Tentative identification	MS/MS fragments	Tags	References
Alkaloids								
P_2074/ P_2073	0.746	180.142	[M + H]^+^	C₁₁H₁₇NO	*Tecomanine	84, 165	M_DCM, M_EAC, D_DCM	^40^
P_2189/ P_2188	1.967	184.1701	[M + H]^+^	C₁₁H₂₁NO	*Hydroxyskytanthine	166,123, 81	M_DCM, SKOV3	^41^
P_1760 P_1759/ P_1758	2.004	166.159	[M + H]^+^	C₁₁H₁₉N	*Dehydroskytanthine;	81, 123	SKOV3, A549	^42^
P_2187/ P_2190	2.871	184.1709	[M + H]^+^	C₁₁H₂₁NO	*Tecostanine	166, 123, 58	M_DCM, A549, SKOV3	^40^
P_1074/ P_1073	3.36	134.0971	[M-H2O + H]^+^	C₉H₁₃NO	*N-methylphenylethanolamine	118, 119, 91	A549, SKOV3, M_DCM	CHEBI, HMDB, COCONUT, FooDB
P_2010	3.37	178.0875	[M + H]^+^	C₁₀H₁₁NO₂	Hydroxytryptophol	132, 160	M_HEX, M_DCM, M_EAC, SKOV3, A549	^43^
P_2069	3.548	180.1023	[M + H]^+^	C₁₀H₁₃NO₂	Fusaric Acid	162, 134	M_DCM, M_EAC, M_TOT	HMDB, CHEBI, COCONUT
P_4521	5.587	284.165	[M + H-H2O]^+^	C₁₈H₂₃NO₃	Isoxsuprine	121, 164	A549	CHEBI, COCONUT, HMDB
P_6416	5.739	352.1758	[M + H]^+^	C₁₆H₂₇NO₆	Europine	264	A549, SKOV3	CHEBI, COCONUT
P_2892	6.683	218.1181	[M + H]^+^	C₁₃H₁₅NO₂	(benzyliminomethyl) pentane dione	171	A549, SKOV3,	COCONUT, PubChem
3676	7.332	403.1141	[M-H]^−^	C₁₉H₂₀N₂O₈	methyl [carbamoyl(methoxy dihydro-benzodioxinyl)ethyl] hydroxy oxo-dihydropyridine carboxylate	283, 359, 309	D_DCM, D_EAC	COCONUT, PubChem
P_3219	8.244	232.1347	[M + H]⁺	C₁₄H₁₇NO₂	Ethyl (Indolyl) methylpropanoate	204, 188, 159	A549, SKOV3	COCONUT, PubChem
P_5590	8.282	322.2016	[M + H]⁺	C₁₈H₂₇NO₄	Butoxyphenyl methylamino-oxo-heptanoic acid	146, 134, 87, 232	A549, SKOV3	COCONUT, PubChem
P_6183	8.847	344.1854	[M + H]⁺	C₂₀H₂₅NO₄	Codamine	162, 134, 178	A549	CHEBI, COCONUT, HMDB, FooDB
P_5146	9.162	306.1489	[M + H]⁺	C₂₀H₁₉NO₂	Benzosimuline	197	A549, SKOV3,	COCONUT, HMDB
P_6516	9.96	356.2224	[M + H]⁺	C₂₂H₂₉NO₃	[Hydroxy[(hydroxy phenyl ethyl) methyl piperidyl]ethyl]phenol	236, 310	A549, SKOV3	COCONUT, PubChem
P_9911	10.476	492.2591	[M + H]⁺	C₂₆H₃₇NO₈	Siwanine B	318, 288	A549, SKOV3	^44^
3851	10.895	415.1513	[M–H]⁻	C₂₁H₂₄N₂O₇	Oxopropaline A	383, 309, 283	A549, SKOV3	COCONUT
P_6883	10.915	370.2013	[M + H]⁺	C₂₂H₂₇NO₄	Corydaline	148, 176, 107	A549, SKOV3	^45^
P_6140/ P_6137	11.633	342.2064	[M + H]⁺	C₂₁H₂₇NO₃	*Caesanine A/B	296, 268, 249	A549, SKOV3	COCONUT
2955	11.725	352.1192	[M–H]⁻	C₂₀H₁₉NO₅	Parfumine	293, 278, 308	A549, SKOV3, D_HEX, D_DCM, D_EAC	GNPS, CHEBI, COCONUT
P_6635	12.304	360.2535	[M + H]⁺	C₂₂H₃₃NO₃	Napelline	204, 176	A549, SKOV3	CHEBI, COCONUT
P_8471	12.362	431.2263	[M + H]⁺	C₁₈H₂₆N₁₀O₃	Netropsin	297	A549, SKOV3	COCONUT
P_7833	12.711	406.2954	[M + H]⁺	C₂₄H₃₉NO₄	Mirabilene isonitrile	190, 232	A549, SKOV3	COCONUT
Amino acids and peptides								
P_736	0.732	116.0719	[M + H]⁺	C₅H₉NO₂	Proline	70	M_TOT	^6^
P_3667	4.711	252.1595	[M + Na]⁺	C₁₄H₂₁NO₃	methyl -(benzyloxyamino)-methyl-pentanoate	116, 178, 137	A549	COCONUT, PubChem
P_3621/ 1575	5.791	250.144	[M + H]⁺	C₁₄H₁₉NO₃	*amino [hydroxy ( methylbutenyl)phenyl]propanoic acid	71, 58, 162	A549, SKOV	GNPS, COCONUT, CHEBI
P_6744	6.905	365.2071	[M + H]⁺	C₁₉H₂₈N₂O₅	N-(N-((Benzyloxy)carbonyl)-L-leucyl)-L-valine	236, 348	A549, SKOV3	PubChem
P_8905	10.886	448.269	[M + H]⁺	C₂₅H₃₇NO₆	[(hydroxy methoxy trimethyl-oxopentadeca trienyl) oxodihydropyrrolyl]acetic acid	274, 244, 218	A549, SKOV3	COCONUT, PubChem
5703	10.901	532.2402	[M–H]⁻	C₂₅H₃₅N₅O₈	Oxyplicacetin	170, 124, 374	A549, SKOV3	PubChem
P_8825	12.699	445.2408	[M + H]⁺	C₂₃H₃₂N₄O₅	Radiosumin B	311	A549, SKOV3	COCONUT, PubChem
Carbohydrate								
1280	1.544	221.0663	[M + H]^−^	C₈H₁₄O₇	Ethyl glucuronide	75	D_HEX, D_DCM, D_EAC	^46^
Fatty acids								
3095	7.821	363.1654	[M–H]⁻	C₁₆H₂₈O₉	hydroxy{[trihydroxy-(hydroxymethyl)oxanyl]oxy}decenoic acid	59, 72, 75, 135	D_HEX, D_DCM, D_EAC	GNPS, COCONUT
3856	7.911	415.1972	[M–H]⁻	C₂₀H₃₂O₉	Ethyl epihydroxyjasmonate glucoside	161, 120	D_DCM, D_EAC	^47^
3129	8.703	365.1825	[M–H]⁻	C₁₆H₃₀O₉	O-hexosyl dihydrodecanoic Acid	105, 147, 71	D_HEX, D_DCM, D_EAC	COCONUT, PubChem
2428	8.842	315.1448	[M–H]⁻	C₁₅H₂₄O₇	Glucosyl dimethyl heptadienoate	97, 193, 151	A549, SKOV3	COCONUT, HMDB, FooDB
2249	9.552	301.1653	[M–H]⁻	C₁₅H₂₆O₆	Glycerol tributyrate	143, 71	A549, SKOV3, D_HEX, D_DCM, D_EAC	NIST
2273	9.746	303.1828	[M–H]⁻	C₁₅H₂₈O₆	Dihydroxypropanoyl hydroxydodecanoate	58, 215, 173, 155	M_DCM, M_EAC	PubChem
2888	9.751	347.1711	[M–H]⁻	C₁₆H₂₈O₈	Hydroxy[(hydroxydecoxy) oxoethyl]butanedioic acid	303, 185, 215, 231	D_EAC	PubChem
3341	9.837	379.1975	[M–H]⁻	C₁₇H₃₂O₉	octanoyl (beta-D-galactosyl)-sn-glycerol	105, 75	D_HEX, D_DCM	Lipidmaps, CHEBI, PubChem
2272	10.069	303.1819	[M–H]⁻	C₁₅H₂₈O₆	Anhydro -O-nonanoylhexitol	215, 173, 183	D_EAC	PubChem
3243	10.809	373.1863	[M–H]⁻	C₁₈H₃₀O₈	Isocucurbic acid hexoside	81, 118, 93, 141	D_DCM	COCONUT, CHEBI
1394	11	231.1601	[M–H]⁻	C₁₂H₂₄O₄	Dihydroxydodecanoic acid	140, 138, 185	A549, SKOV3	^48^
2594	11.199	327.2179	[M–H]⁻	C₁₈H₃₂O₅	Trihydroxyoctadeca dienoic acid	211, 229, 171	A549, SKOV3	^49^
P_4577	11.249	286.2013	[M + H]⁺	C₁₅H₂₇NO₄	Tert-Butoxycarbonylamino-4-cyclohexylbutyric acid	130, 268, 83, 69	A549, SKOV3	PubChem
2295	11.374	305.1966	[M–H]⁻	C₁₅H₃₀O₆	Xylitol decanoate	58, 155	SKOV3	PubChem
P_4288	12.127	275.1855	[M + H]⁺	C₁₄H₂₆O₅	(Hydroxyoctanoyloxy)hexanoic acid	55, 59, 69, 57	A549, SKOV3	PubChem
P_7473	12.276	392.2795	[M + H]⁺	C₂₃H₃₇NO₄	Dioctyl Pyridine dicarboxylate	176, 218	A549, SKOV3	PubChem
P_4673/ P_4677	12.37	289.2009	[M + H]⁺	C₁₅H₂₈O₅	*Decyl hydroxypentanedioic acid	55, 59, 67, 57	A549, SKOV3	^50^
P_4290	12.373	275.1856	[M + H]⁺	C₁₄H₂₆O₅	(hydroxyundecyl)propanedioic acid	83	A549, SKOV3	COCONUT, PubChem
P_3479	12.375	243.1592	[M + H]⁺	C₁₃H₂₂O₄	Octylpent enedioic acid	69, 57, 71, 83	A549, SKOV3	^51^
1891	12.474	275.1866	[M–H]⁻	C₁₄H₂₈O₅	Trihydroxytetradecanoic acid	72, 132, 229	A549, SKOV3, D_HEX, D_DCM	PubChem
P_4676	12.698	289.201	[M + H]⁺	C₁₅H₂₈O₅	methyl acetoxy hydroxy-dodecanoate	55, 69, 81, 87	A549, SKOV3	COCONUT
P_9918	13.697	492.4058	[M + H]⁺	C₃₀H₅₃NO₄	Tricosa trienoylcarnitine	318, 162	A549, SKOV3	HMDB, PubChem
2131	14.219	293.2133	[M–H]⁻	C₁₈H₃₀O₃	Hydroxyoctadeca trienoic acid (HOTre)	275, 235, 223 ,195	M_HEX	^52^
P_4357	14.22	277.2179	[M + H]⁺	C₁₈H₂₈O₂	Stearidonic acid	259, 135, 93, 81	M_HEX, D_HEX	^11^
P_6456	14.378	353.2706	[M + H]⁺	C₂₁H₃₆O₄	Monolinolenin	261, 95, 81, 67	M_HEX	^53^
6089/6088	14.859	555.2903	[M–H]⁻	C₂₅H₄₈O₁₁S	* α-Sulfoquinovosyl monoacylglyceride	299, 225, 207, 81	M_HEX, M_DCM	^54^
Polyketides								
1026	5.307	197.0821	[M-H]^−^	C₁₀H₁₄O₄	Dimethyl octadienedioic acid	71, 179, 68	D_HEX, D_DCM, D_EAC	PubChem
P_2742	5.408	211.133	[M + H-H2O]^+^	C₁₂H₂₀O₄	Dibutyl maleate	95, 71, 67, 68	A549	COCONUT
Shikimates and Phenylpropanoids								
79	3.165	109.0294	[M–H]⁻	C₆H₆O₂	Catechol	59	D_EAC	^55^
1949	4.124	281.0668	[M–H]⁻	C₁₃H₁₄O₇	Feruloyl Lactate	161, 135, 139, 59	D_EAC	PubChem
P_1091	4.518	135.0804	[M + H]⁺	C₉H₁₀O	Acetophenone	65, 79, 91	A549	NIST, CHEBI, COCONUT
P_2100	5.316	181.086	[M + H]⁺	C₁₀H₁₂O₃	Peniciphenol	163, 91, 93, 115	A549, SKOV3	^56^
750	5.43	173.0457	[M–H]⁻	C₇H₁₀O₅	Shikimic acid	111, 93, 83	D_EAC	^57^
P_1682	5.504	163.0764	[M + H]⁺	C₁₀H₁₀O₂	Safrole	91, 65, 77, 119	M_TOT	CHEBI, COCONU, HMDB
3712/3711	5.511	405.1419	[M + FA]⁻	C₁₉H₂₁NO₆	*Feruloylnormetanephrine	197, 71, 359, 179	M_TOT	^58^
P_1414	5.545	149.0971	[M + H]⁺	C₁₀H₁₂O	Anethole	91,93, 121, 119	M_DCM	CHEBI, COCONUT, HMDB
2005 /P_4598	9.629	285.0439	[M–H]⁻	C₁₅H₁₀O₆	*Luteolin	133, 151, 217	M_EAC	^59^
2709	6.095	335.0774	[M–H]⁻	C₁₆H₁₆O₈	Trihydroxy (oxoisochromenyl)oxy-cyclohexanecarboxylic acid	161, 161, 93	D_EAC	COCONUT
2207/ P_5012	11.2	299.0577	[M–H]⁻	C₁₆H₁₂O₆	Caffeoylshikimic acid	284, 256, 284	M_DCM, M_EAC, D_DCM, D_EAC	^59^
2708	6.226	335.0772	[M–H]⁻	C₁₆H₁₆O₈	Scroside D	173, 161, 93, 255	D_EAC	^60^
7472	6.81	653.2109	[M–H]⁻	C₃₀H₃₈O₁₆	Acteoside	161, 179, 151, 133	M_EAC	^61^
7066	7.017	623.201	[M + H]⁺	C₂₉H₃₆O₁₅	Umbelliferone	161, 133, 71, 135	M_HEX, M_DCM, M_EAC, M_TOT	^10^
P_1671	7.021	163.0398	[M–H]⁻	C₉H₆O₃	Hemiphroside A	135, 117, 145	M_EAC	^62^
7687	7.473	667.2263	[M–H]⁻	C₃₁H₄₀O₁₆	Isoacteoside	621, 179	M_EAC	COCONUT, PubChem
7065	7.479	623.2	[M–H]⁻	C₂₉H₃₆O₁₅	Nitrophenol	161, 135, 113, 71	M_EAC, M_TOT	^10^
375	7.802	138.0198	[M + H]⁺	C₆H₅NO₃	Trihydroxy-dimethoxy flavone	108, 69	A549, SKOV3, DCM	Massbank
P_5331	7.956	313.0706	[M–H]⁻	C₁₇H₁₄O₇	Ferulic acid	298, 303	A549, SKOV3	COCONUT, PubChem
984	8.199	193.051	[M–H]⁻	C₁₀H₁₀O₄	Benzofuranol	134, 133, 161	A549, SKOV3	^63^
310	8.202	133.0296	[M–H]⁻	C₈H₆O₂	Methoxy cinnamic acid	105, 89	SKOV3	COCONUT, PubChem
803	9.853	177.056	[M–H]⁻	C₁₀H₁₀O₃	Ethyl caffeate	117, 89	A549, SKOV3	CHEBI, COCONUT, FooDB, HMDB
1135	9.912	207.0668	[M–H]⁻	C₁₁H₁₂O₄	*Apigenin	161, 134	A549, SKOV3	^64^
1816/ P_4136	10.835	269.0475	[M–H]⁻	C₁₅H₁₀O₅	*Chrysoeriol	251, 149, 117, 151	M_EAC	^59,65^
P_8152	11.474	419.1856	[M + H]⁺	C₂₆H₂₆O₅	gangetinin	359, 331	A549, SKOV3	CHEBI, COCONUT, PubChem
966	11.632	191.0714	[M–H]⁻	C₁₁H₁₂O₃	p-Coumaric acid ethyl ester	163, 117, 145	A549, SKOV3	^66^
3430	12.071	385.1504	[M–H]⁻	C₁₈H₂₆O₉	Juniperoside	127, 71, 139	A549, SKOV3, D_HEX, D_DCM	^67^
Terpenoids								
1958	5.547	281.1393	[M–H]⁻	C₁₅H₂₂O₅	Epidihydrophaseic Acid	123, 171	D_EAC	CHEBI, COCONUT, FooDB, HMDB
3461	12.697	387.1658	[M–H]⁻	C₁₈H₂₈O₉	Epihydroxyjasmonic acid hexoside	127, 331, 72	A549, SKOV3	CHEBI, COCONUT, FooDB, HMDB
3048	5.307	359.1346	[M–H]⁻	C₁₆H₂₄O₉	Deoxyloganic acid	197, 71, 211	D_HEX, D_DCM, D_EAC	CHEBI, COCONUT
2889	10.889	347.1712	[M–H]⁻	C₁₆H₂₈O₈	Kankanoside N	99, 73, 72	D_HEX, D_DCM, D_EAC	COCONUT, PubChem
2890	10.35	347.1712	[M–H]⁻	C₁₆H₂₈O₈	Foeniculoside VII	72, 285, 73, 55	D_EAC	COCONUT, PubChem
3271/3269	10.059	375.1659	[M–H]⁻	C₁₇H₂₈O₉	Ajureptoside	99, 72, 154, 229	D_EAC	PubChem
3052	10.236	359.1494	[M–H]⁻	C₂₀H₂₄O₆	Muricarpone A	127, 187, 125	D_DCM, D_EAC	COCONUT
P_14413	5.505	743.2749	[M + Na]⁺	C₃₂H₄₈O₁₈	Pratialin B	383, 203, 221, 383	M_TOT	COCONUT, PubChem
5220	12.982	503.337	[M–H]⁻	C₃₀H₄₈O₆	Madecassic acid	485	A549	COCONUT, PubChem
P_1150	8.966	137.1328	[M–H₂O + H]⁺	C₁₀H₁₈O	Citronellal	81, 95, 67	A549	^68^
P_1194	9.848	139.1484	[M + H]⁺	C₁₀H₁₈	Camphane	55, 83, 69, 57	A549, SKOV3	CHEBI, COCONUT, HMDB
P_1549	9.848	157.1594	[M + H]⁺	C₁₀H₂₀O	D-Citronellol	83, 69, 55, 57	A549, SKOV3	^68^
P_2466	7.297	197.1184	[M + H]⁺	C₁₁H₁₆O₃	Loliolide	179, 197, 135, 107	A549, SKOV3	CHEBI, COCONUT, HMDB
P_3641	5.406	251.1256	[M + H]⁺	C₁₄H₁₈O₄	Helinorbisabone	188	A549	PubChem
3218	12.222	371.2078	[M–H]⁻	C₁₉H₃₂O₇	Blumenol C glucoside	261, 253	A549, SKOV3	PubChem
4961	10.287	487.1818	[M + Cl]⁻	C₂₂H₃₂O₁₂	methyl diacetoxy methyl [trihydroxy-(hydroxymethyl)tetrahydropyranyl] oxyhexahydroindene carboxylate	127, 83, 154, 55	D_HEX	COCONUT, PubChem
5065	5.752	493.2284	[M + FA]⁻	C₂₂H₃₈O₁₂	Rhodioloside	59, 87, 55, 71	D_HEX	COCONUT, PubChem
5628/ 5627	7.633	527.2371	[M + FA]⁻	C₂₂H₃₈O₁₃	*hexosyl-hexosyl-geraniol	72, 75, 135, 57	M_HEX, M_TOT	PubChem
P_9498	13.107	474.3215	[M + H]⁺	C₂₈H₄₃NO₅	Deoxyoxysporidinone	340, 164	A549, SKOV3	COCONUT, PubChem
P_10715	9.646	529.1884	[M + H]⁺	C₂₄H₃₂O₁₃	Tecoside	367,157,119	SKOV3, A549	

Significance of the tags:

P: denotes for metabolites identified in the positive ion mode.

asterisk (*): denotes for the elution of different isomeric form of the metabolite.

M: Denotes for a major metabolite.

D: Denotes for a discriminatory metabolite.

SKOV3/A549: Denote for the metabolites with potential cytotoxicity against SKOV3 or A549 cell lines.

HEX: Denotes for the *n*-hexane fraction.

DCM: Denotes for the dichloromethane fraction.

EAC: Denotes for the ethyl acetate fraction.

### IC₅₀ values for total extract and fractions

The cytotoxic potential of the *T. stans* total extract (TOT) and its fractions; *n*-hexane (HEX), dichloromethane (DCM) and ethyl acetate (EAC), against SKOV-3 and A549 cell lines was assessed using the MTT assay (Table 1). All fractions demonstrated measurable cytotoxicity, with SKOV-3 cells displaying greater sensitivity than A549 cells. Among the tested fractions, DCM exhibited the highest cytotoxic activity against SKOV-3 cells [IC₅₀ value of 18.50 µg/mL (95% CI: 17.93–19.11)] and A549 cells [IC₅₀ value of 21.95 µg/mL (95% CI: 20.07–24.00)]. The HEX fraction also demonstrated notable yet weaker cytotoxicity, with IC₅₀ values of 71.58 µg/mL (95% CI: 66.28–77.30) and 83.11 µg/mL (95% CI: 79.89–86.47) against SKOV-3 and A549, respectively. In contrast, EAC and TOT showed comparatively higher IC₅₀ values, indicating lower potency. Overall, SKOV-3 consistently exhibited greater susceptibility to *T. stans* fractions compared with A549 cell line (Table 2) (Fig. S3 and Fig S4).

**Table 2:** The IC_50_ values for the crude extract fractions, positive control (doxorubicin) and CRY on SKOV-3 and A549 cell lines (all values are expressed in µg/ml).

Cell line	HEX	DCM	EAC	TOT	CRY	Doxorubicin
IC_50_ SKOV-3 (95% CI)	71.58 (66.28–77.30)	18.50 (17.93–19.11)	121.62 (115.2-128.6)	486.80 (468.1-506.2)	30.14 (25.23–35.99)	0.023 (0.019–0.029)
IC_50_ A549 (95% CI)	83.11 (79.89–86.47)	21.95 (20.07-24.00)	226.63 (218-235.5)	720.29 (677.7-765.5)	43.88 (34.46–55.91)	0.036 (0.027–0.048)

### LC–MS/MS driven multivariate data analysis of *T. stans* extracts and bioactive fractions

The initial UPLC-QTOF-MS/MS analysis of the three bioactive fractions (HEX, DCM, EAC) and the total 70% ethanol extract detected approximately 3,800 metabolites, of which 1,099 were validated as statistically significant against blank controls. A biochemometric multivariate analysis was then applied to refine biologically relevant markers.

**Fig. 1:**
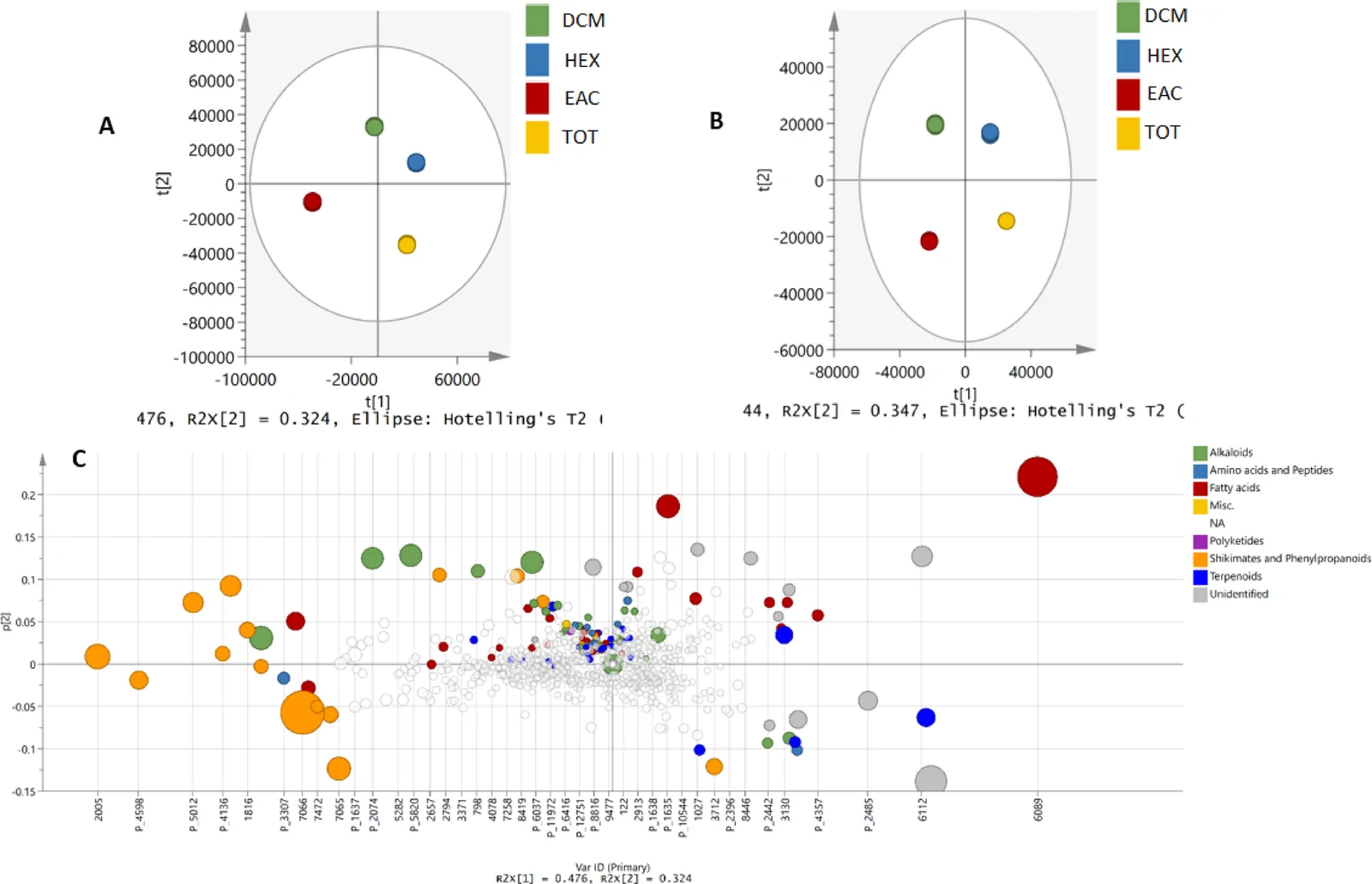
Principal component analysis (PCA) of Pareto scaled UHPLC-MS features of *T. stans* extract and fractions (**A**) Score plot of statistically significant 1099 features against blank, (**B**) Score plot of the selected features, (**C**) loading plot coloured by chemical classes and features were labelled according to the alignment ID with “P_” prefix for ESI+ detected features and sized according to the sum of features abundance across samples. NA; denotes statistically significant features against blank but not major, differential nor correlated to the bioactivity.

Unsupervised principal component analysis (PCA) of the full 1,099 feature dataset revealed distinct chemical profiles for the total extract and its fractions (Fig. 1A). Notably, reducing the dataset to a refined subset of discriminant features preserved the major variance between samples (Fig. 1B), confirming that the feature reduction strategy effectively retained metabolites contributing to differentiation. Inspection of the PCA loading plots identified several key metabolites driving sample separation. The DCM fraction was primarily characterized by alkaloids such as *hydroxyskytanthine* (P_2188) and *tecomanine* (P_2073), along with the sulfolipid fatty acid derivative *α-SQMG* (6089). In contrast, separation of the ethyl acetate and total extract clusters was influenced by flavonoids including *luteolin* (P_4598) and *apigenin* (P_4136), while the n-hexane fraction was distinguished by fatty acids such as *stearidonic acid* (P_4357). Together, these loading plot features highlight key metabolites contributing to the chemical differentiation among fractions (Fig. 1C).

**Fig. 2:**
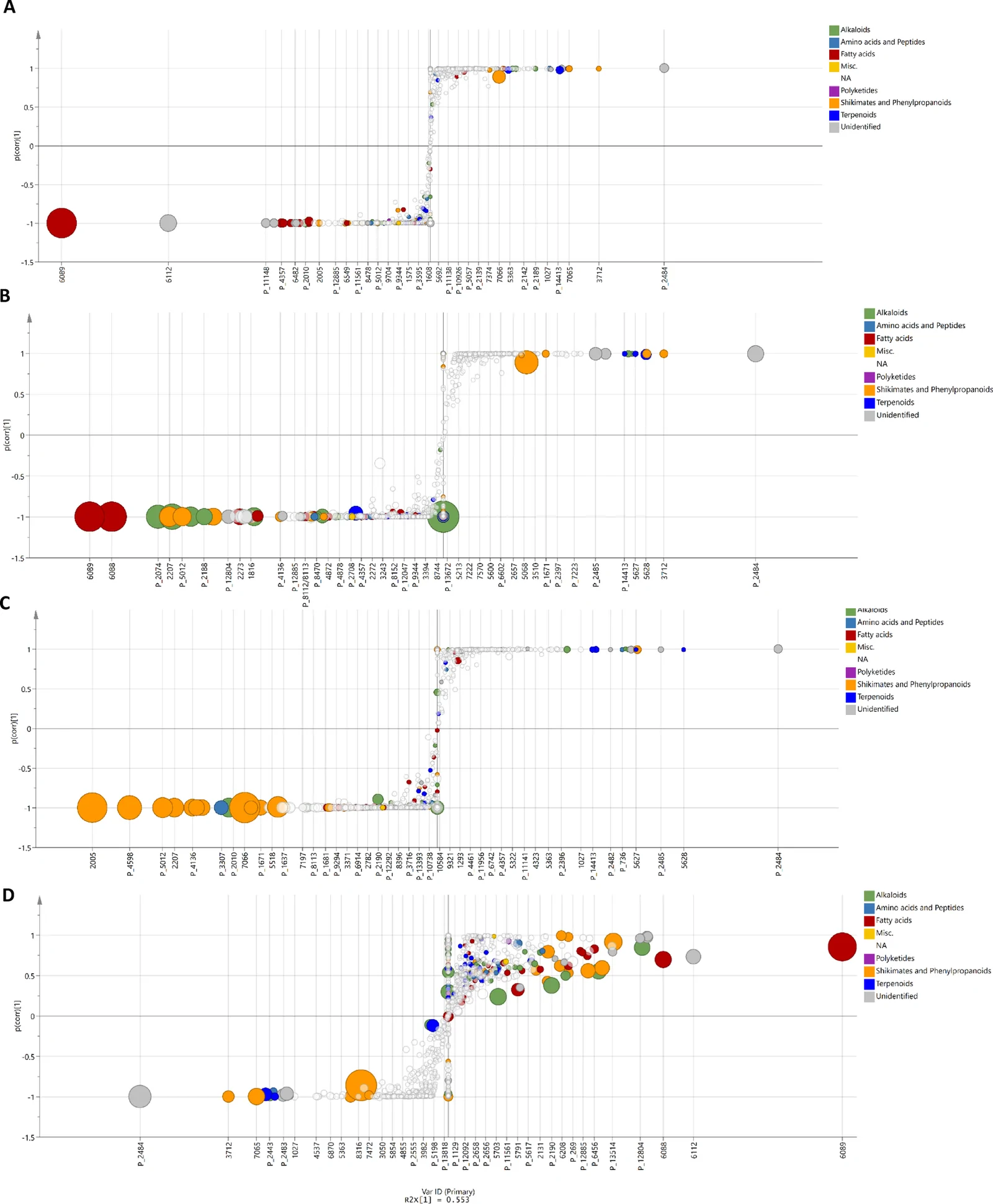
Orthogonal partial least square analysis showing loading scatter S-Plot of Pareto scaled UHPLC-MS significant 1099 features of different extracts vs. TOT and compounds are coloured by chemical classes. (**A**) HEX vs. TOT features are sized by average abundance in HEX (**B**) DCM vs. TOT features are sized by average abundance in DCM, (**C**) EAC vs. TOT features are sized by average abundance in EAC and (**D**) DCM + HEX vs. TOT features are sized by sum abundance in all samples.

Supervised orthogonal partial least squares-discriminant analysis (OPLS-DA) was applied to further refine the dataset and identify the most discriminatory metabolites unique to each fraction (Fig. 2). Pairwise OPLS-DA S-plot analyses between each fraction and the total extract enabled the identification of fraction-enriched features (Fig. 2A–C). Additionally, a combined OPLS-DA S-plot analysis was conducted on the fractions with the highest bioactivity (HEX and DCM) versus the total extract to identify shared discriminatory metabolites (Fig. 2D).

To establish a link between metabolite abundance and cytotoxic activity, a bioactivity correlation analysis was conducted using bioactive molecular networking workflow that was established by Nothis et al. 2018 ^17^. This approach correlated metabolite abundance with IC₅₀ values against SKOV3 and A549 cancer cell lines, which led to the identification of 63 bioactivity linked metabolites among the 107 identified compounds. These bioactive compounds were labeled with “SKOV3” and/or “A549” tags, signifying their potential contribution to cytotoxic activity against the corresponding cell line (Table 2). The corresponding correlation scores for each metabolite–activity relationship are provided in Supplementary table S1.

### Discriminatory, major and key bioactive metabolites in each fraction

In the HEX Fraction the predominant discriminatory metabolites were primarily fatty acids. α-Sulfoquinovosyl monoacylglyceride (α-SQMG) (6088 and 6089) and Stearidonic acid (P_4357) exhibited the highest discriminatory effect (Fig. 2A). The fraction demonstrated lower abundance of shikimates and terpenoids.

The OPLS-DA S-plot of the DCM fraction (Fig. 2B) revealed that alkaloids, fatty acids and flavonoids were the predominant metabolite classes, exhibiting high relative abundance and strong discriminatory effects. Among the most abundant and highly discriminatory metabolites were α-SQMG (6088 and 6089), Tecomanine (P_2073 and P_2074), CRY (2207 and P_5012), and Hydroxyskytanthine (P_2188).

The EAC fraction was characterized by a high abundance of shikimates, particularly flavonoids. The principal discriminatory metabolites included luteolin (2005, P_4598), CRY (2207 and P_5012), and apigenin (1816, P_4136), all of which were detected in both positive and negative ionization mode where the metabolite ID preceded with ”P_” are detected in ESI+ mode. The fraction exhibited a relatively low abundance of terpenoids, alkaloids, and fatty acids (Fig. 2C).

The S-plot derived from the combined data of the most bioactive fractions (HEX and DCM) (Fig. 2D) identified α-SQMG (6088 and 6089) as the most discriminant and major metabolite in both fractions, suggesting its potential correlation with cytotoxic activity.

### Isolation and cytotoxic evaluation of chrysoeriol (CRY)

Given that the DCM fraction exhibited the highest cytotoxic activity against both SKOV-3 and A549 cell lines, it was selected for further fractionation. Chromatographic separation of the DCM fraction led to the isolation of CRY as a pale yellow powder (428 mg). The detailed isolation procedure is described in the Materials and Methods section.

The chemical structure of CRY was identified by comparing its spectral data, including mass spectrometry (MS), ¹H-NMR, and ^13^C-NMR, with those previously reported in the literature^59,65^. CRY was identified in this study as a major discriminative metabolite of the DCM fraction based on the metabolomic and biochemometric analyses performed (Fig. 2.B and Table 2).

The cytotoxic potential of CRY against SKOV-3 and A549 cells were further evaluated. CRY displayed cytotoxicity against SKOV-3 [IC₅₀ value 30.14 µg/mL (95% CI: 25.23–35.99)] and A549 [IC_50_ value of 43.88 µg/mL (95% CI: 34.46–55.91)]. However, doxorubicin displayed a markedly higher potency against both cell lines, with IC₅₀ values of 0.023 µg/mL (95% CI: 0.019–0.029) and 0.036 µg/mL (95% CI: 0.027–0.048), respectively (Table 2).

Based on these findings, both DCM and CRY were selected for mechanistic investigations aimed at elucidating the molecular basis of their cytotoxic activity. The SKOV-3 cell line was prioritized for these analyses, given its higher sensitivity, with doxorubicin included as a positive control.

### Standardization of DCM fraction

CRY was subsequently utilized as a chemical marker for standardization of the DCM fraction using UPLC. The chromatogram of the DCM fraction recorded at 345 nm (Fig. S5) displayed a prominent peak at a retention time (Rt) of 12.66 min, corresponding to CRY. The UPLC chromatogram of the isolated reference compound is shown in (Fig. S6).

Linearity was assessed by preparing a series of CRY standard solutions at concentrations of 31.25, 62.5, 125, 250, and 500 µg/mL. A calibration curve was generated by plotting peak area versus concentration (Fig. S7), demonstrating excellent linearity over the tested range. The regression equation was y = 0.3954x + 0.3771, with a correlation coefficient of R² = 0.9999.

Quantitative analysis indicated that 1.0 g of the DCM fraction contained 41.8 mg of CRY. Validation parameters for the UPLC method including linearity range, accuracy, limit of detection (LOD), limit of quantification (LOQ), and precision are summarized in (Table S2).

### Mechanistic cytotoxicity assessment

#### Cell cycle analysis

**Fig. 3:**
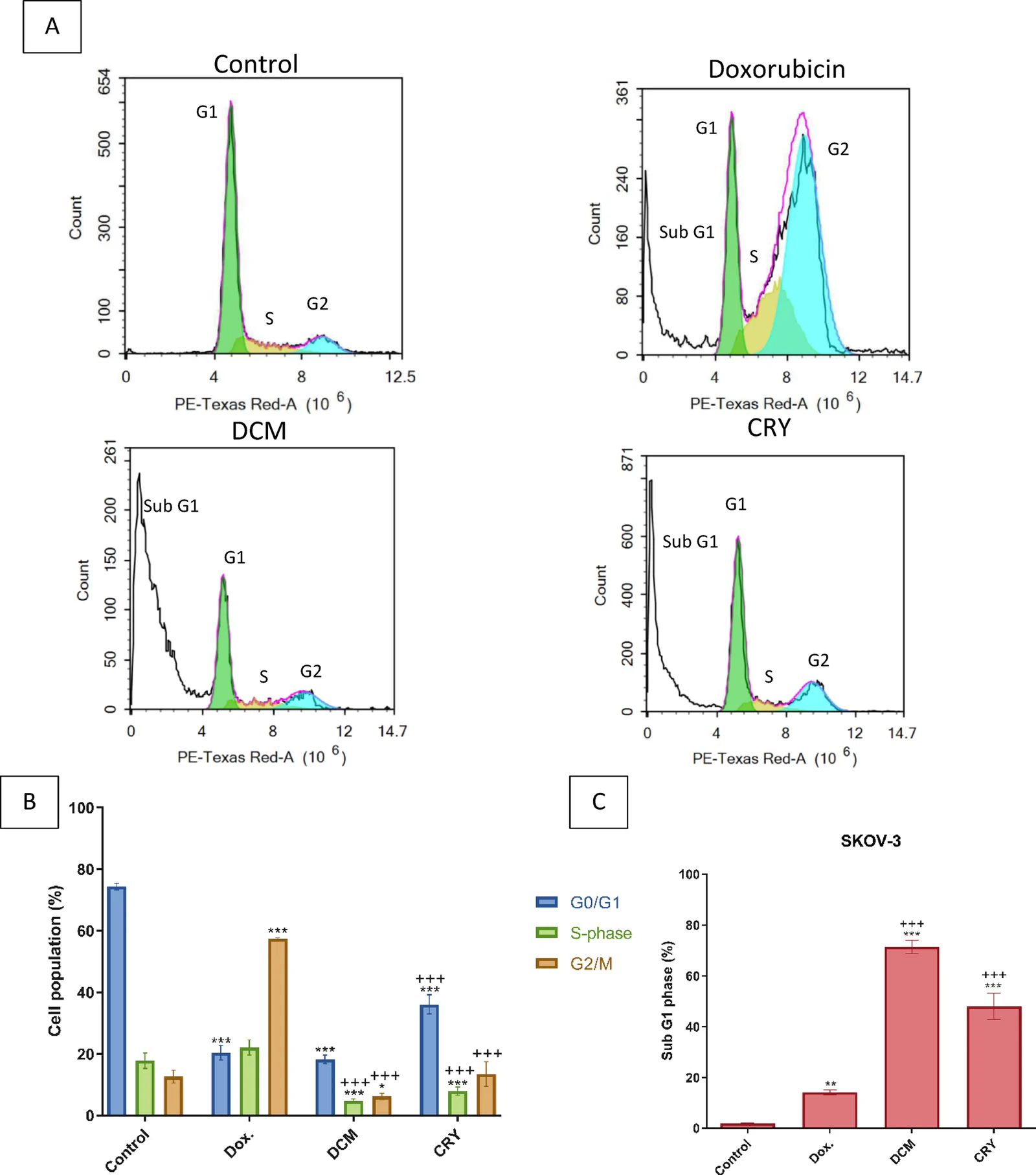
Cell cycle analysis of SKOV-3 cells treated with the IC₅₀ concentrations of the DCM, CRY, doxorubicin and negative control. (**A**): Representative histograms showing the distribution of cells amongst the various cell cycle phases. (**B**): Quantification of the percentage of cells in each phase of the cell cycle is depicted as a bar chart showing the mean (*n* = 3) and error bars are for standard deviation. (**C**): Sub-G1 phase was plotted as percentage of total events. Statistical analysis was done using one-way ANOVA followed by Tukey’s post hoc. Where * is statistically significant difference from the control and + is statistically significant difference from Doxorubicin (*** :*P* < 0.0005, **: *P* < 0.005, *: *P* < 0.05, +++:*P* < 0.0005).

The effects of the standardized DCM fraction and CRY on SKOV-3 cell cycle progression were evaluated in comparison to vehicle treated control and the positive control doxorubicin. Flow cytometric analysis quantified the distribution of cells across G₀/G₁, S, and G₂/M phases, as well as the sub-G₁ fraction, which reflects apoptotic DNA fragmentation (Fig. 3).

Treatment with the DCM fraction induced a marked accumulation of cells in the sub-G₁ phase (71.42 ± 2.63%), significantly exceeding all other groups (*P* < 0.0005). This was accompanied by a reduction in the G₀/G₁ population to 18.21 ± 1.37% and decrease in the S and G₂/M phases population to 4.74 ± 0.68% and 6.18 ± 1.02% respectively.

CRY treatment also significantly increased the sub-G₁ population to 48.02 ± 5.22% *P* < 0.0005 vs. controls), with concomitant reductions in G₀/G₁, S and G₂/M phases population to 36.08 ± 3.07%, 7.91 ± 1.32% and 13.48 ± 4.02% correspondingly.

Doxorubicin treatment primarily induced G₂/M arrest, with an average of 57.37 ± 0.38% of cells in this phase, significantly higher than control, DCM and CRY (*P* < 0.0005). This was associated with an S-phase population of 22.10 ± 2.46% and a relatively low sub-G₁ fraction (14.19 ± 0.93%) as compared to both DCM and CRY (*P* < 0.0005).

#### Annexin V/Propidium iodide (PI) apoptosis assay

**Fig. 4:**
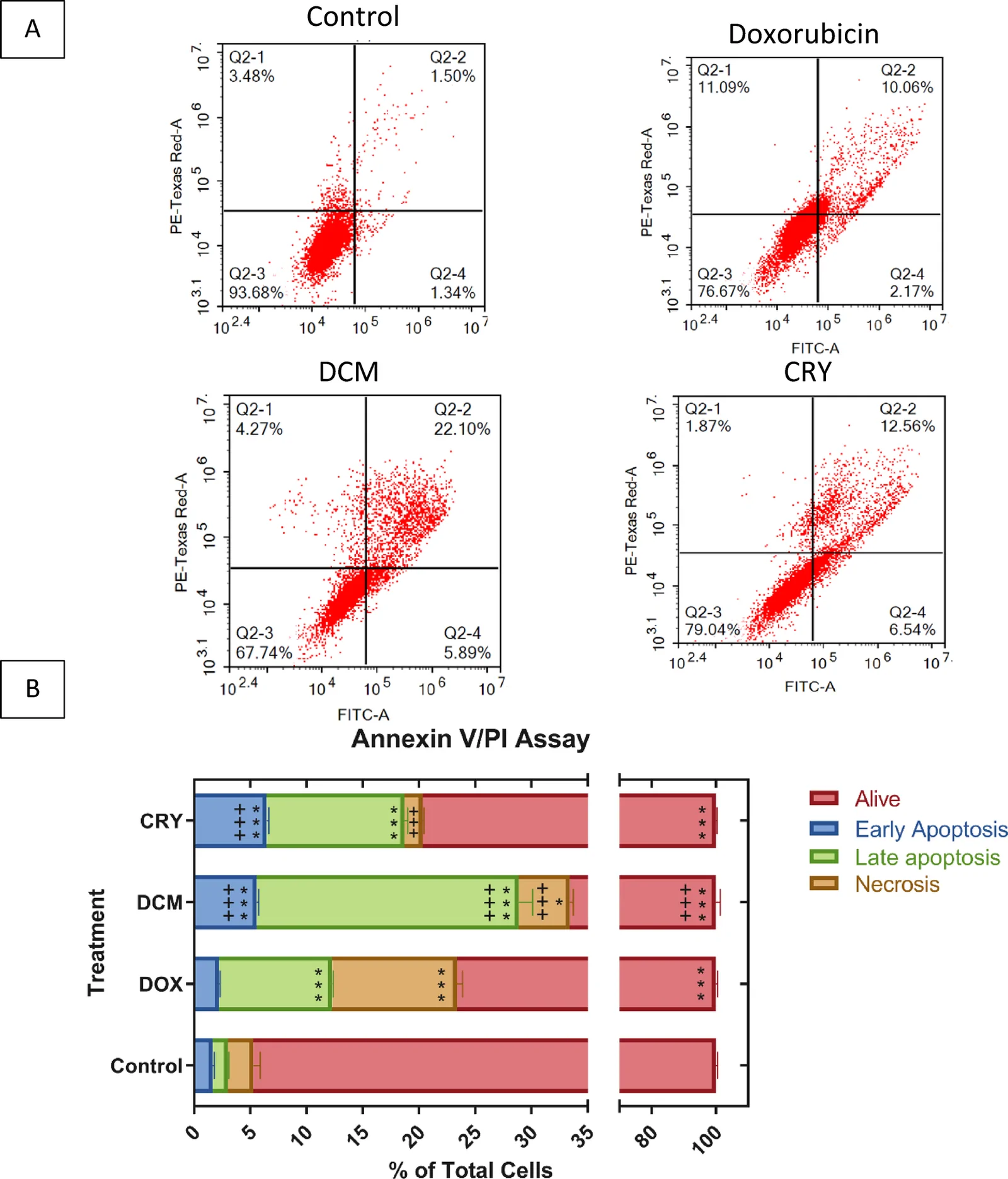
Apoptosis assessment of SKOV-3 cells treated with the IC₅₀ concentrations of the DCM, CRY, doxorubicin and negative control *via* Annexin V/PI assay. (**A**): representative dot plot showing the distribution of gated intact cell population after exclusion of subcellular events amongst necrosis (Q2-1), late apoptosis (Q2-2), early apoptosis (Q2-4) and intact (viable) cells (Q 2–3). (**B**): stacked bar chart showing the percentage of cells in each stage. Values represent mean (*n* = 3) and error bars are for SD. Statistical analysis was done using one-way ANOVA followed by Tukey’s post hoc. Where * is statistically significant difference from the control and + is statistically significant difference from doxorubicin (*** :*P* < 0.0005, *: *P* < 0.05, +++:*P* < 0.0005).

The apoptotic response of SKOV-3 cells following treatment with the DCM fraction and CRY was assessed using Annexin V/PI dual staining, with untreated cells and doxorubicin serving as negative and positive controls, respectively. Flow cytometric analysis enabled discrimination of viable (Q3), early apoptotic (Q4), late apoptotic (Q2), and necrotic (Q1) populations (Fig. 4).

Exposure to the DCM fraction resulted in a marked induction of apoptosis, characterized by a significant increase in the late apoptotic population to 23.32 ± 2.10% significantly exceeding both control groups (*P* < 0.0005), this was accompanied by a reduction in viable cells to 66.59 ± 2.38%. Necrotic and early apoptotic populations remained relatively low at 4.52 ± 0.53% and 5.56 ± 0.32%, respectively, confirming apoptosis as the predominant mode of cell death.

CRY treatment also promoted apoptosis, with late apoptotic cells comprising 12.22 ± 0.53% of the population significantly higher than the negative control (*P* < 0.0005). Although this effect was less pronounced than that of the parent fraction, it was associated with a reduction in viability to 79.70 ± 0.59%, while necrotic and early apoptotic populations represented 1.61 ± 0.23% and 6.47 ± 0.29%, respectively.

Treatment with doxorubicin showed a viable and late apoptotic population of 76.64 ± 0.90% and 10.06 ± 0.17% that wasn’t significantly different from the CRY. With significantly lower early apoptotic population 2.21 ± 0.17% and higher necrotic population, 11.08 ± 0.90% as compared to DCM and CRY (*P* < 0.0005).

### Cell migration assay

**Fig. 5:**
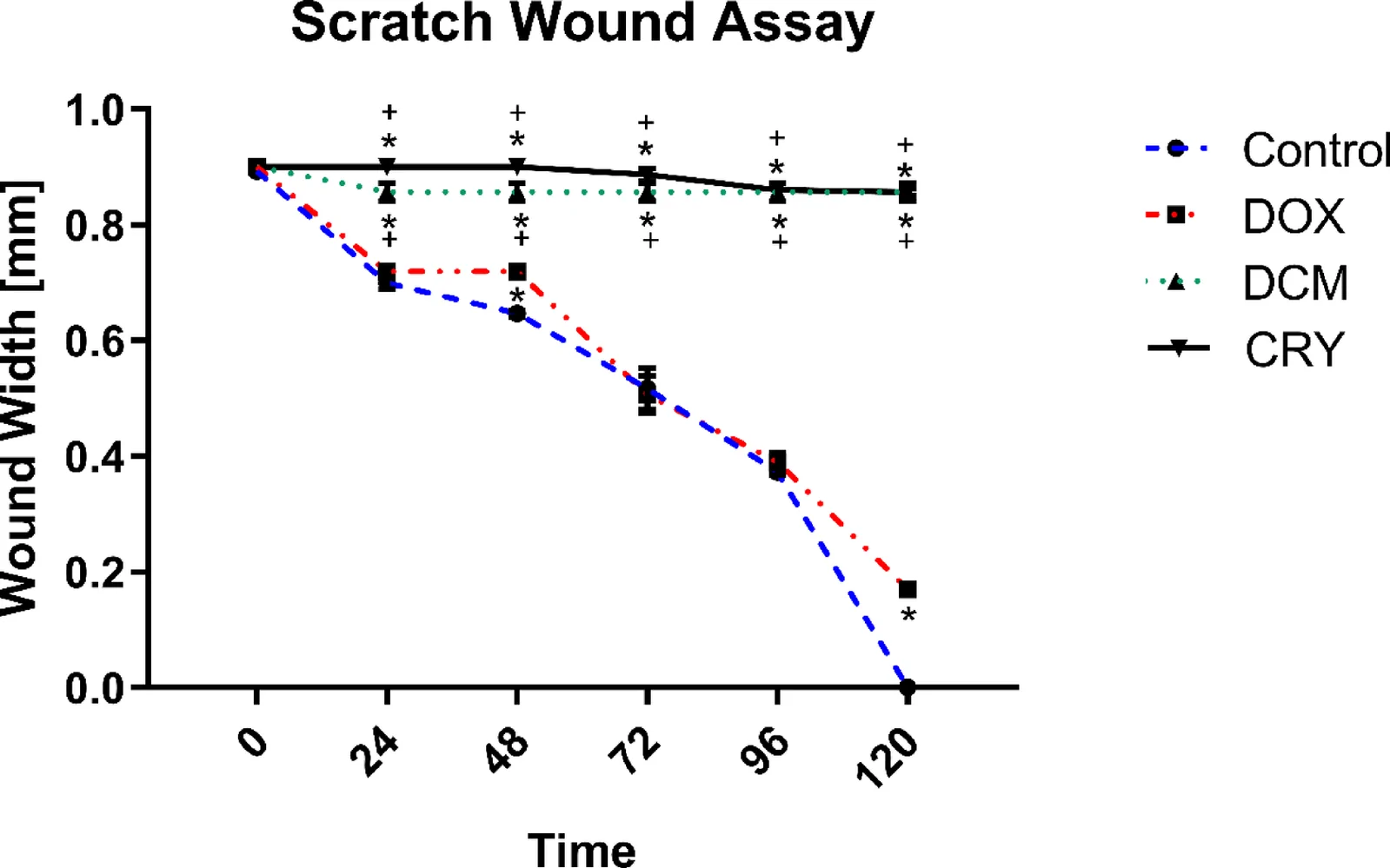
Line chart representing wound width (mm) over time (0–120 h) in SKOV-3 cells treated with IC₅₀ concentrations of doxorubicin, DCM fraction, CRY and control. Statistical analysis was performed using two-way ANOVA followed by Tukey’s post hoc test. Where * is statistically significant difference from the control and + is statistically significant difference from doxorubicin * :*P* < 0.0001, +:*P* < 0.0001.

**Fig. 6:**
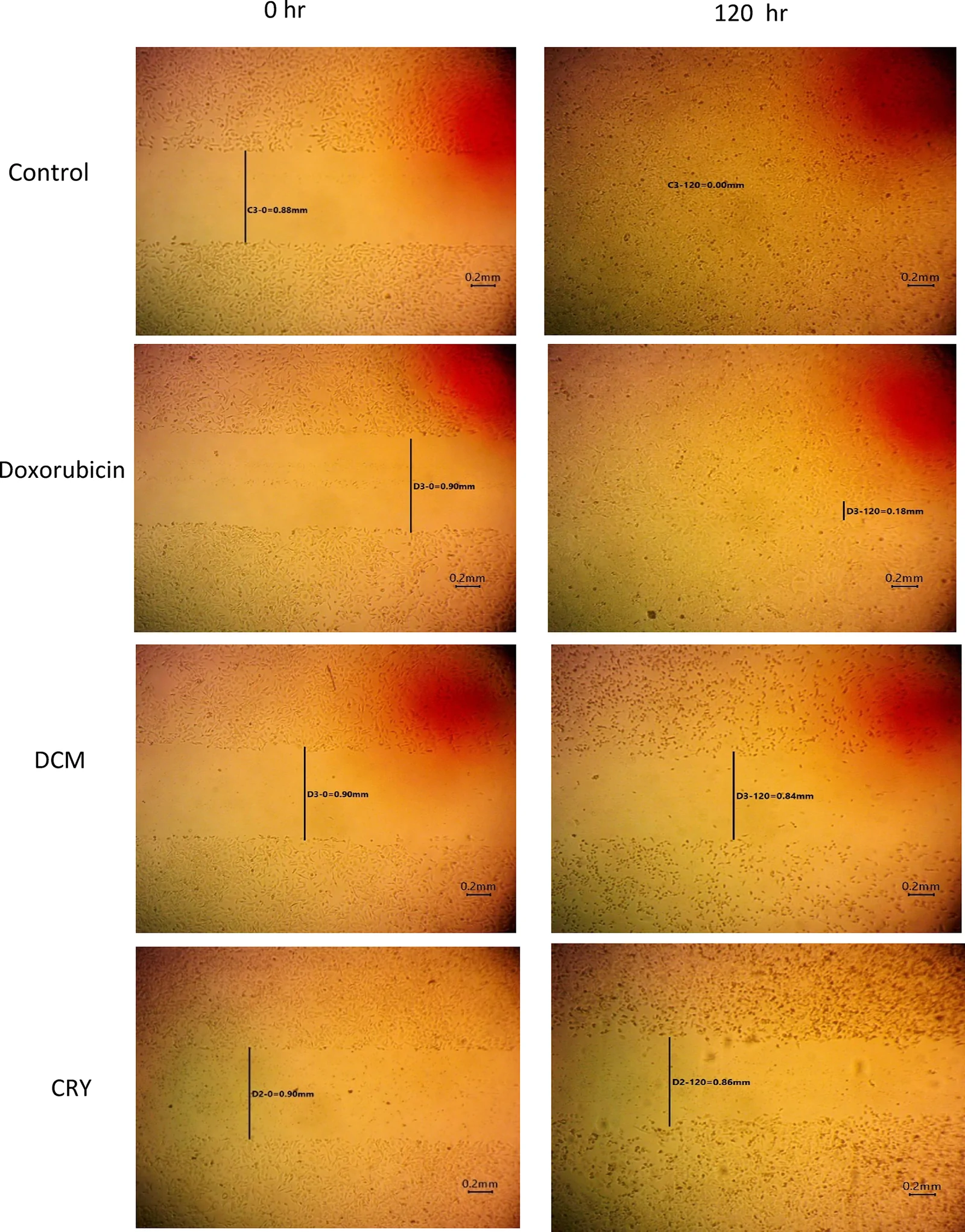
Selected photomicrograph showing the wound closure of the SKOV-3 cells treated with Doxorubicin, DCM, and CRY at 0 h and 120 h.

The wound healing assay was performed to assess the impact of the DCM fraction, CRY, and doxorubicin on SKOV-3 cell migration over 120 h. A defined linear scratch was introduced into confluent monolayers, and wound closure was measured at set intervals as an indicator of migratory capacity (Figs. 5 and 6).

Doxorubicin treated cells demonstrated progressive wound closure, with wound width constantly decreasing from 0.90 mm at baseline to 0.17 mm at 120 h, exhibiting no significant difference between the positive and negative control except at the 48 h and 120 h time intervals (Fig. S9).

In contrast, the DCM fraction significantly reduced wound closure at all time points compared to control and doxorubicin treated cells (*p* < 0.0005). Wound width showed minimal change, decreasing from 0.90 mm to 0.86 mm at 24 h and remaining constant through 48, 72, 96, and 120 h, reflecting sustained suppression of wound healing (Fig. S10).

Similarly, CRY treatment displayed marked and sustained reduction in wound closure, with wound width maintained at 0.90 mm throughout the first 72 h and only slight reductions observed at 96 h (0.89 mm) and 120 h (0.86 mm). This inhibitory effect was statistically significant relative to both control and doxorubicin treated groups at all time points assessed (*p* < 0.0005) (Fig. S11).

### ELISA quantification of apoptosis- and metastasis-related proteins

#### Caspase-3

**Fig. 7:**
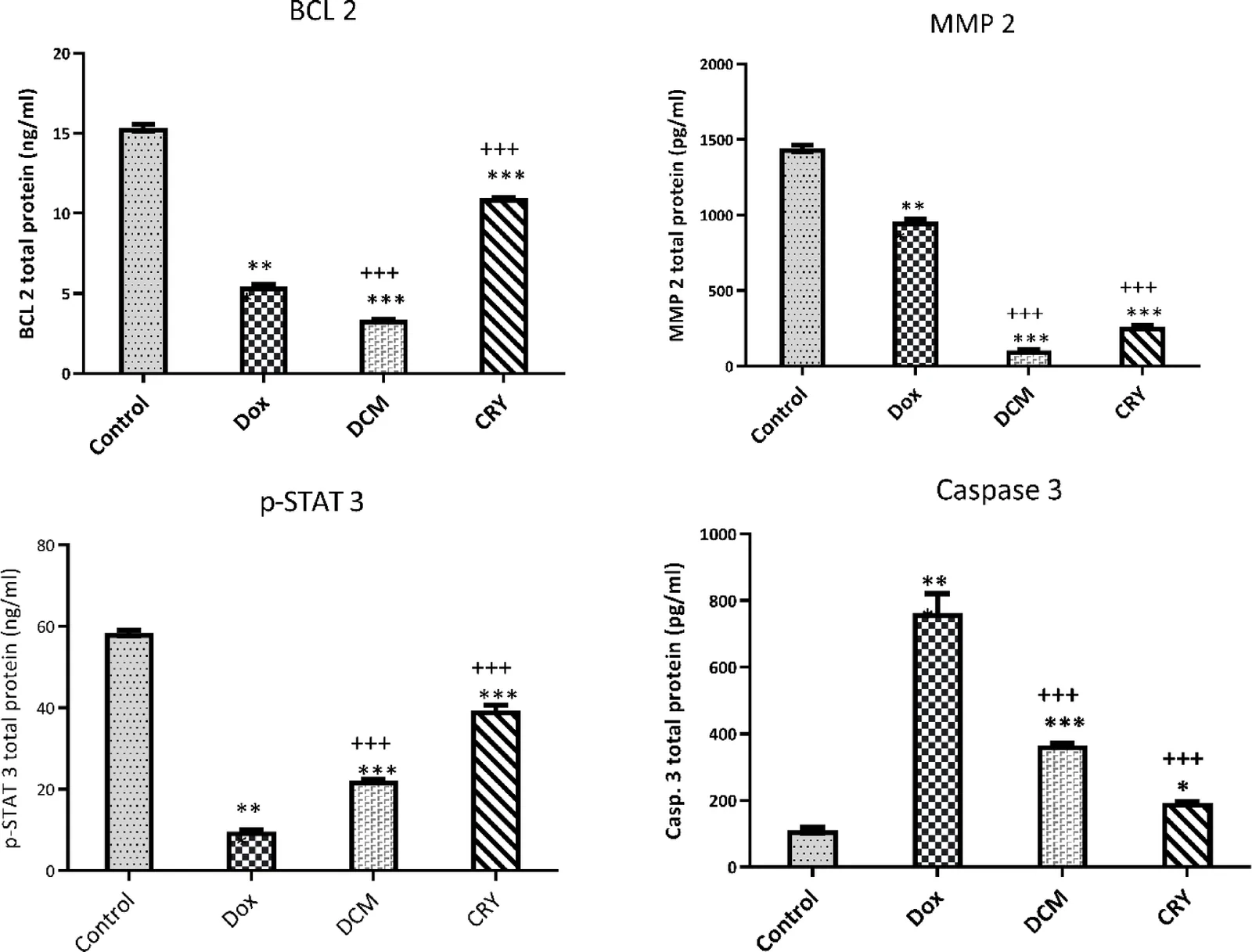
Caspase 3, p-STAT3, BCL 2 and MMP 2 ELISA results on SKOV-3 following treatment with doxorubicin, DCM fraction and CRY. Values represent mean (*n* = 3) and error bars are for SD. Statistical analysis was done using one-way ANOVA followed by Tukey’s post hoc. Where * is statistically significant difference from the control and + is statistically significant difference from doxorubicin (*:*P* < 0.05,*** :*P* < 0.0005, +++:*P* < 0.0005).

Measurement of caspase-3, a key executioner caspase in apoptosis, revealed significant elevations in its concentration following treatment with the DCM fraction and CRY. The DCM fraction induced an average caspase-3 level of 364.95 ± 6.18 pg/mL, while CRY treatment yielded 192.06 ± 5.56 pg/mL. Both values were significantly higher than that recorded for the untreated control group 110.69 ± 8.54 pg/mL (*P* < 0.05). The most pronounced increase was observed with doxorubicin, which elevated caspase-3 levels to 762.53 ± 58.36 pg/mL, a value significantly greater than all other treatment groups (*P* < 0.0001) (Fig. 7).

#### p-STAT3

Evaluation of phosphorylated STAT3 levels demonstrated marked suppression by both the DCM fraction and CRY compared to negative control cells (*P* < 0.0001). DCM treated cells exhibited an average p-STAT3 concentration of 22.20 ± 0.23 ng/mL, while CRY treatment resulted in 39.35 ± 1.34 ng/mL, both significantly lower than the control group mean of 58.36 ± 0.71 ng/mL. Doxorubicin treatment showed the highest inhibitory effect, reducing p-STAT3 to 9.64 ± 0.45 ng/mL markedly lower than the other treatment groups (*P* < 0.0001).

#### Bcl-2

Quantification of the anti apoptotic protein Bcl-2 indicated that all tested treatments significantly downregulated its expression relative to control cells (*P* < 0.0001). The DCM fraction produced the most substantial reduction, with an average concentration of 3.37 ± 0.05 ng/mL, followed by doxorubicin at 5.43 ± 0.16 ng/mL. CRY treated cells showed higher Bcl-2 levels at 10.96 ± 0.03 ng/mL than the other treatments, yet still significantly lower than the untreated control at 15.33 ± 0.22 ng/mL.

#### MMP-2

Assessment of matrix metalloproteinase-2 expression revealed strong inhibitory effects by both the DCM fraction and CRY. DCM reduced MMP-2 to an average of 104.65 ± 6.09 pg/mL, while CRY treatment yielded 261.69 ± 10.47 pg/mL; both were significantly lower than the control value of 1441.67 ± 20.71 pg/mL (*P* < 0.0001). Doxorubicin also decreased MMP-2 expression, with an average of 958.07 ± 16.83 pg/mL, though this effect was significantly lower than that of DCM or CRY (*P* < 0.0001).

## Discussion

The current study explored the cytotoxic potential of *T. stans* leaf extract and fractions against SKOV-3 and A549 cell lines, identified metabolites potentially associated with this activity and elucidated the mechanistic basis of the cytotoxic activity against SKOV-3, the most sensitive cell line. DCM fraction exhibited the highest potency, with substantially lower IC₅₀ values compared to the crude extract and other fractions. Fractionation of DCM yielded CRY, a flavonoid previously reported from *T. stans*^2,12^. CRY showed measurable cytotoxicity against both SKOV-3 and A549 cells, with SKOV-3 exhibiting greater sensitivity. To our knowledge, this represents the first evidence of the cytotoxic effect of CRY against ovarian carcinoma cells, thereby extending its pharmacological relevance.

The DCM was standardized to contain 41.8 µg CRY per mg of the DCM fraction, corresponding to 4.18% (w/w). The LC–MS/MS driven metabolomic profiling revealed a diverse chemical composition of alkaloids, fatty acids, flavonoids, terpenoids and phenolics across the fractions. The biochemometric analysis further prioritized 63 candidate metabolites potentially associated with cytotoxic activity. Notably, the analysis showed that the DCM fraction, which exhibited the highest cytotoxic activity, was characterized by the presence of key major and/or discriminatory metabolites including α-SQMG (6088 and 6089), CRY (2207 and P_5012), and tecomanine (P_2073 and P_2074), 5-hydroxytryptophol (P_2010), 7α-hydroxyskytanthine (P_2188) and N-methylphenylethanolamine (P_1074). These results stand in line with previous reports regarding the cytotoxic activity of both CRY^69,70^ and α-SQMG^71^, supporting their possible contribution to the activity of the DCM fraction. The candidate metabolites identified through correlation filtering were also supported by OPLS-DA-based multivariate analysis, although further validation in studies with a greater number of fractions would improve the robustness of metabolite activity assignment.

Elucidation of the cytotoxic mechanism was performed through a series of cellular and molecular assays against SKOV 3 cell line after treatment with DCM, CRY and doxorubicin as a positive control. the results showed that DCM and CRY primarily exerted their cytotoxic effects through apoptosis induction where the Flow cytometric analysis revealed a substantial accumulation of SKOV-3 cells in the sub-G₁ phase, which indicates extensive DNA fragmentation and confirms apoptotic induction at the genomic level^72^, in contrast to doxorubicin, which induced G₂/M arrest which aligns with its known ability to interfere with mitotic progression^73^.

The apoptotic nature of cell death was further confirmed using Annexin V/PI staining. DCM fraction induced a marked increase in the late apoptotic population, with minimal necrosis or early apoptosis, indicating a well-regulated apoptotic cascade. DCM demonstrated more moderate but significant late apoptotic action, while maintaining relatively low levels of necrosis and early apoptosis. In comparison, doxorubicin produced a mixed apoptotic necrotic profile, with elevated necrosis and moderate levels of apoptosis, suggesting a broader spectrum of cytotoxic stress mechanisms.

In addition to apoptosis induction, the results of the scratch wound healing assay showed a marked reduction in wound closure following treatment with DCM and CRY, which may suggest a potential anti migratory activity. While cytotoxicity addresses primary tumor reduction, impaired wound closure may also be relevant to the migratory behavior of ovarian cancer cells, which typically disseminates via the peritoneal fluid^74^. The downregulation of MMP-2 observed in this study provides a biochemical rationale for this effect. As an enzyme responsible for degrading the basement membrane^75^, MMP-2 is a prerequisite for the invasion and distal spread of SKOV-3 cells, which are clinically characterized by their high invasive potential^74^. However, further assessment using sub IC₅₀ concentrations is recommended to more specifically confirm whether the observed reduction in wound closure reflects a direct anti migratory effect.

ELISA based protein quantification of Bcl-2, caspase-3, STAT3 and MMP levels provided supporting evidence for the involvement of apoptosis and metastasis related pathways. The DCM fraction and CRY significantly increased caspase-3 activity, consistent with activation of the execution phase of apoptosis. Both treatments also reduced the expression of p-STAT3, a key transcription factor promoting proliferation, survival, immune evasion and metastasis^76^, and downregulated the anti apoptotic protein Bcl-2. The concurrent activation of caspase-3, suppression of Bcl-2, and inhibition of STAT3 signaling indicates that the observed cytotoxicity is associated, at least in part, with modulation of apoptosis-related and pro-survival signaling markers. Although the ELISA analysis of Bcl-2 and caspase-3 suggests the involvement of apoptotic signaling, additional markers such as BAX and cytochrome c would be required to fully characterize the mitochondrial apoptosis pathway.

The marked reduction in p-STAT3 observed in treated cells was accompanied by lower levels of Bcl-2 and MMP-2, both of which are reported downstream targets of STAT3 signaling^76^. Because STAT3 activation has been implicated in the transcriptional regulation of Bcl-2 and MMP-2, the concurrent reductions observed in these markers may be associated with reduced p-STAT3 levels and are consistent with modulation of STAT3-related survival and migration pathways. However, targeted mechanistic studies, such as STAT3 activation, would be required to confirm a direct causal relationship.

Notably, the activity of the isolated CRY was moderate compared to that of DCM, suggesting that the superior potency of the fraction may reflect the combined or additive contribution of multiple metabolites rather than the effect of CRY alone.

Although published data regarding the mechanistic assessment of *T. stans* cytotoxic activity remain scarce^5^, the available literature supports the findings reported in the current study. For instance, earlier reports demonstrated that the endophytic metabolite of *T. stans* preferentially induced apoptosis over necrosis in MCF-7 cells, consistent with the apoptotic mode of cell death observed in our SKOV-3 model^5^. Similarly, the suppression of Bcl-2 expression following treatment with the DCM fraction and CRY aligns with the findings of Reddy et al. (2021)^4^ who reported downregulation of Bcl-2 mRNA expression in MCF-7 cells treated with *T. stans* leaf extract. Together, these findings not only reinforce the anticancer potential of *T. stans* derived metabolites but also highlight their potential application in targeting metastatic cancer phenotypes.

Despite the promising findings, the present study has certain limitations. First, the absence of a non-tumorigenic control cell line limits the assessment of cancer cell selectivity. The potential combined or additive interactions among the identified active metabolites were not directly investigated, which may contribute to the overall cytotoxic activity of the extract. Moreover, although the obtained data support apoptosis induction, further characterization of apoptosis-related pathways using complementary molecular markers is required for a more comprehensive mechanistic understanding. Future studies should therefore focus on including appropriate non-tumorigenic cell models to assess selectivity and translational relevance, in addition to assessment of the isolated metabolite combination would be required to determine whether the observed effects are synergistic, additive, or independent beside, expanded molecular investigations to further elucidate the therapeutic potential of *T. stans*.

## Conclusion

This study provides the first LC–MS/MS-based metabolomic and biochemometric characterization of the cytotoxic activity of *T. stans* leaves extract and its fractions. The analysis identified several metabolites potentially associated with the observed bioactivity, including chrysoeriol (CRY), α-sulfoquinovosyl monoacylglyceride (α-SQMG), tecomanine, and 7α-hydroxyskytanthine. Biological investigations demonstrated that a standardized *T. stans* fraction and CRY exert significant cytotoxic and wound closure inhibition activity against SKOV-3 ovarian cancer cells, likely mediated through apoptosis induction and modulation of key molecular markers, including caspase-3, Bcl-2, phosphorylated STAT3, and MMP-2. These findings highlight *T. stans* as a promising source of bioactive metabolites with anticancer potential and provide a basis for further mechanistic and in vivo investigations.

Overall, this work provides a valuable foundation for future studies aimed at validating the anticancer potential of *T. stans* and further characterization of apoptosis related pathways using complementary molecular markers.

## Supplementary Information

Below is the link to the electronic supplementary material.


Supplementary Material 1



Supplementary Material 2


## Data Availability

Upon request, the data supporting the results of this article will be made available by the corresponding author.
