# Supplementary material for: Metabolomic and biochemometric profiling of Tecoma stans with mechanistic insights of its in vitro cytotoxic activity
Source: Sci Rep. 2026 Aug 27;16:26940. doi: 10.1038/s41598-026-67317-z (PMC13522503; doi:10.1038/s41598-026-67317-z)
Supplement: Supplementary file 1 [file 41598_2026_67317_MOESM2_ESM.docx]

Supplementary figures

**Figure S2:** The overlaid base peak chromatograms of the crude extract and the bioactive fractions in the negative ion mode

**Figure S1**: The overlaid base peak chromatograms of the crude extract and the bioactive fractions in the positive ion mode

**Figure S3:** The dose response curves following the treatment for 48 hours with the total extract, active fractions, CRY and DOX on SKOV-3 cell line. Each value represents the mean (n=3) and error bars are for standard deviation (SD).

**Figure S4:** The dose response curves following the treatment for 48 hours with the total extract, active fractions, CRY and DOX on A549 cell line. Each value represents the mean (n=3) and error bars are for standard deviation (SD).

**Figure S5**: UPLC chromatogram of *T.stans* DCM fraction

**Figure S6**: UPLC chromatogram of chrysoeriol

**Figure S7**: Calibration curve of chrysoeriol

**Figure S8:** Selected photomicrograph showing the cell migration for the negative control treated SKOV-3 cells throughout time span from 0 hr to 120 hr.

0 hr

24 hr

48 hr

72 hr

96 hr

120 hr

0 hr

24 hr

48 hr

72 hr

96 hr

120 hr

**Figure S9:** Selected photomicrograph showing the cell migration for the doxorubicin treated SKOV-3 cells throughout time span from 0 hr to 120 hr

0 hr

24 hr

48 hr

72 hr

96 hr

120 hr

**Figure S10:** Selected photomicrograph showing the cell migration for the DCM treated SKOV-3 cells throughout time span from 0 hr to 120 hr

0 hr

24 hr

48 hr

72 hr

96 hr

120 hr

**Figure S11:** Selected photomicrograph showing the cell migration for the CRY treated SKOV-3 cells throughout time span from 0 hr to 120 hr

**A. Cell-cycle analysis gating**

| **Control** | **Doxorubicin** |
| --- | --- |
|  |  |
| **CRY** | **DCM** |
|  |  |

**Supplementary Figure S12**: Representative flow cytometry gating strategy

**B. Annexin V/PI apoptosis gating**

| **Control** | **Doxorubicin** |
| --- | --- |
|  |  |
| **CRY** | **DCM** |
|  |  |

**Supplementary Figure S12:** Representative flow cytometry gating strategy (continued)
